# Study on brain functional networks and wearable device monitoring in children with SeLECTs and high spike-wave index (SWI >50%)

**DOI:** 10.3389/fneur.2025.1571330

**Published:** 2025-09-30

**Authors:** Minghao Xu, Xiaoxuan Li, Yifan Fu, Dinghan Hu, Yang Zhang, Wang Wei, Yaotian Gao, Keyi Lin, Bin Yang, Jiuwen Cao, Tao Jiang

**Affiliations:** ^1^Department of Neurosurgery, The First Affiliated Hospital of Anhui Medical University, Hefei, Anhui, China; ^2^Anhui Public Health Clinical Center, Hefei, Anhui, China; ^3^Machine Learning and I-Health International Cooperation Base of Zhejiang Province, and the Artificial Intelligence Institute, Hangzhou Danzi University, Zhejiang, China; ^4^Department of Neurosurgery, The First Affiliated Hospital of Anhui University of Traditional Chinese Medicine, Hefei, Anhui, China; ^5^Department of Neurology, Anhui Province Children's Hospital, Hefei, Anhui, China

**Keywords:** SWI, ACC, PPG, EDA, DTF, SeLECTs

## Abstract

**Introduction:**

Self-limited epilepsy with centrotemporal spikes (SeLECTs) represents a common idiopathic focal epilepsy syndrome in childhood. Although most patients demonstrate a favorable prognosis, some patients develop ESES. ESES is associated with poorer neuropsychological prognosis. This association challenges the “benign” classification of SeLECTs. Currently, the diagnostic threshold for ESES remains controversial. Moreover, traditional long-term video-EEG monitoring presents certain limitations.

**Methods:**

The research utilizes the “Biovital-P1” software integrated with Oppo smart bands to collect multimodal physiological signals. Simultaneously, a 21-channel digital EEG system acquires electroencephalographic data. The study constructs brain networks through DTF analysis. Additionally, it performs preprocessing and feature extraction on multimodal physiological signals (ACC, EDA, PPG).

**Results:**

The results demonstrate strong functional connectivity in the centrotemporal region in all frequency bands and the delta band. However, as SWI levels increase, the brain network's global and local efficiency significantly reduces. Analyzing multimodal physiological signals reveals statistically significant differences in ACC and PPG signal time-domain features (Maximum, Minimum, Peak) among different SWI groups. Multiple characteristic parameters of EDA signals also show significant intergroup differences. Notably, EDA signals exhibit excellent sensitivity in reflecting stress responses of the autonomic nervous system. The characteristic features of EDA signals demonstrate a significant negative correlation with SWI levels.

## 1 Introduction

Self-limited epilepsy with centrotemporal spikes (SeLECTs) represents the most common idiopathic focal epilepsy syndrome in childhood (1). Traditionally, this condition, formerly known as “benign epilepsy with central temporal lobe spikes,” has been considered to have a favorable prognosis, with most patients in remission by the age of 16 (2). In epidemiologic surveys, SeLECTs is the most common form of self-limited focal epilepsy, accounting for 6–7% of all childhood epilepsies. Its incidence is about 6.1 cases per 100,000 children under 16 years of age per year (3). The most distinctive feature of SeLECTs is the presence of variable spike discharges in the Rolandic region during interictal periods. Notably, these discharges' quantity significantly correlates with disease severity (4). However, some patients develop electrical status epilepticus during sleep (ESES), which complicates the clinical picture. ESES is frequently associated with poorer neuropsychological prognosis, manifesting as significant cognitive deficits, intellectual decline, and behavioral problems (5). These associations challenge the traditional classification of SeLECTs as a “benign” condition.

ESES is characterized by generalized spike-and-wave complexes at 1.5–3.0 Hz during non-rapid eye movement (NREM) sleep, demonstrating continuous or near-continuous discharges (6, 7). The Spike and Wave Index (SWI) is a crucial diagnostic criterion for ESES. In 1971, Patry et al. (8) first identified ESES and established that a diagnosis requires three consecutive monthly recordings showing SWI between 85% and 100%. Subsequent studies have revealed that patients with SWI ranging from 50% to 65% exhibit relatively better performance in cognitive function tests. When SWI falls below 85%, the impact on cognitive impairment appears less significant. Currently, significant discrepancies exist among researchers regarding the diagnostic threshold for ESES. These differences reflect the complexity and diversity of ESES diagnosis. Different diagnostic thresholds may lead to substantially varied diagnoses for the same patient, potentially affecting treatment decisions and prognosis evaluation. Furthermore, this inconsistency presents considerable challenges for establishing unified diagnostic criteria through future research.

Clinical studies demonstrate that in SeLECTs, higher SWI values correlate with increased epileptic activity and more significant abnormalities in brain electrical activity, elevating the risk of neuropsychological complications (9). Patients with discharge indices ≥50% exhibit prolonged P300 latency, reduced amplitude, and lower cognitive test scores compared to those with lower discharge indices (10). These findings indicate intellectual impairment in SeLECTs patients, with the severity increasing with more frequent electroencephalogram(EEG) discharges. Correlation analysis confirms negative relationships between EEG discharge index and both P300 parameters and intelligence test scores.The most common cognitive deficits in children with SeLECTs are attention deficits (selective attention, impulse control) and language dysfunction (vocabulary extraction and comprehension), which are both directly related to active epileptic phases, and their early identification and targeted interventions can improve the long-term prognosis (11, 12). Therefore, traditional EEG alone cannot comprehensively assess patient conditions, necessitating additional cognitive function evaluations. Furthermore, the substantial workload associated with long-term video-EEG monitoring poses significant challenges for clinicians. This monitoring method requires considerable time and human resources while impacting patients' daily lives. Consequently, identifying a simplified and effective alternative approach has become crucial for clinical practice.

In summary, significant controversy remains regarding the diagnostic threshold of spike-wave index (SWI) in SeLECTS, and the mechanisms by which epileptiform discharges impair brain function are not yet fully understood. Conversely, traditional long-term video-EEG monitoring, though widely utilized, imposes several limitations with regard to accessibility, comfort, and continuous tracking in real-life settings.

**Figure 1:**
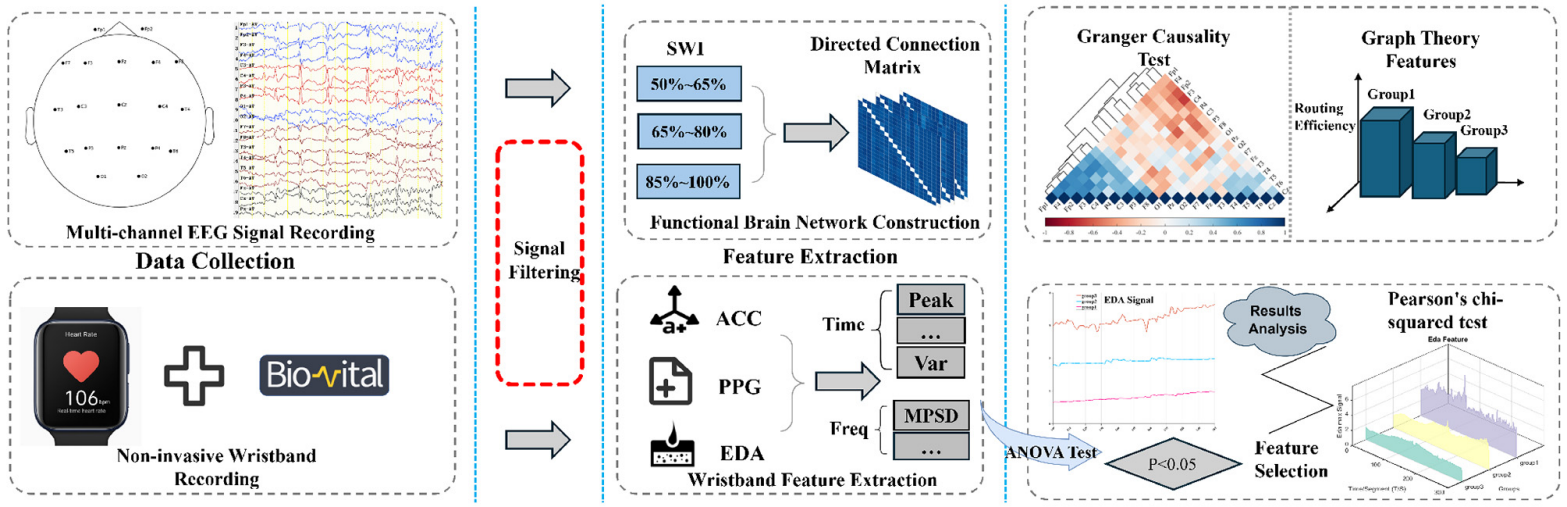
The flowchart comprises three distinct sections to systematically illustrate the methodology. The leftmost section delineates the instrumentation and raw data acquisition process. Subsequently, the central section elaborates on the signal extraction procedures for EEG, PPG, ACC, and EDA modalities. The concluding section presents the statistical analytical framework employed for result interpretation. EEG, Electroencephalogram; PPG, Photoplehysmography; ACC, Three-axis acceleration; EDA, Electrodermal activity.

The primary objective of this study is to investigate the intrinsic mechanisms of SWI-related brain function impairment in SeLECTS from a neurophysiological signal perspective, while also evaluating the potential of portable monitoring devices as a practical and non-invasive alternative to conventional EEG.To achieve this objective, we first collected electroencephalogram (EEG) and multimodal physiological data from 15 patients diagnosed with SeLECTS. The participants were stratified into three groups based on their SWI levels ( 50%−65%, 65%−80%, and >85%), with the objective of enabling a comprehensive examination of the differential impact of discharge burden on brain function. Subsequently, by comparing traditional electroencephalogram (EEG) monitoring with physiological signals acquired from portable devices, the feasibility and accuracy of wearable technologies in SeLECTS monitoring were assessed. Finally, we employed brain network analysis and multimodal feature extraction techniques to explore changes in brain functional connectivity and neurophysiological signals. As shown in Figure 1, the research framework comprises three main components: data collection and preprocessing, brain network construction and analysis, and multimodal physiological signal feature extraction with statistical analysis.

## 2 Materials and methods

### 2.1 Patients

All patient data were collected from Anhui Provincial Children's Hospital, strictly adhering to medical ethics standards. Informed consent was obtained from all patients' guardians, and the study fully complied with institutional ethical regulations. Data were collected between 8 April 2021 and December 2023, encompassing 432 epilepsy patients aged 3–13 years, representing various epilepsy types. Each patient wore an Oppo smart band featuring “Biovital-P1” software for physiological signal acquisition. The device integrates a three-axis accelerometer and gyroscope for effective data collection. EEG signals were recorded using 32 electrodes positioned according to the 10–20 system (Fp1, Fp2, F3, F4, C3, C4, P3, P4, O1, O2, F7, F8, T3, T4, T5, T6, Fz, Cz, Pz, etc.). Linked mastoids (M1/M2) served as the online reference and AFz as the ground. ECG was monitored using chest electrodes, and two EMG channels (submental and tibialis anterior muscles) tracked muscle activity. Signals were sampled at 500 Hz (EEG), 200 Hz (ECG/EMG), and were bandpass-filtered (EEG: 0.1–70 Hz; ECG: 1–40 Hz; EMG: 10–200 Hz). EEG monitoring lasted 16 h, and sleep-wake phases were scored in 30-s cycles based on American Academy of Sleep Medicine (AASM) criteria. Total sleep time and wake duration were quantified from EEG/electroencephalography patterns. The recording protocol included both wakefulness and sleep states, with a minimum of 4 h dedicated to sleep state recording for the comprehensive capture of brain activity across different states. The following inclusion criteria were applied for SeLECTs patient selection:

1. The age range of SeLECTs patients was 6–13 years.
2. SWI exceeded 50%.
3. Patients had no history of stroke or neuropsychiatric disorders and had not taken antiepileptic drugs in the past three months.
4. Patients exhibited no motor dysfunction and had normal cognitive function before epilepsy onset.

Clinical EEG experts accurately marked seizure periods and SWI by visual inspection. SWI, defined as the percentage of spike-wave discharges per unit time, serves as a key indicator for assessing the severity of abnormal EEG discharges in SeLECTs patients. Research has demonstrated that SWI is closely associated with seizure frequency, cognitive impairment, and disease prognosis (13), with a higher SWI indicating more frequent abnormal discharges and the development of more severe neurological dysfunction (14). Grouping patients according to SWI therefore facilitates a deeper understanding of disease severity and its specific impacts on patients. Studies have shown that SeLECTs patients with SWI>50% often exhibit more significant cognitive impairment, while those with SWI >85% face a higher risk of seizures. Patients in this study were divided into three SWI interval groups: SWI 50%−65%(Group 1), SWI 65%−80% (Group 2), and SWI >85% (Group 3). Ultimately, five patients were in the SWI 50%−65% group, seven patients in the SWI 65%−80% group, and three patients in the SWI >85% group. This categorization facilitates a more detailed analysis of the correlation between abnormal discharge levels and physiological signals recorded by wristbands and provides more precise data for subsequent analyses. Detailed patient information is presented in Table 1.

**Table 1:** Demographics detailed information.

**Characteristic**	**Patients with SeLECTs**	***p*-values**
**Demographics**		
Age(y)	7.733 ± 1.279	0.632
Sex(M/FM)	7/8	0.523
**DI (Discharge index)**		
>50%	15	
50%−65%	5	
65%−80%	7	
85%−100%	3	
**History of epilepsy**		
Age at epilepsy onset(y)	5.867 ± 1.798	
Time to last seizure(m)	0.516 ± 0.467	
Types of antiepileptic drugs	2 ± 1	
Frequency of seizures at recording	0.467 ± 0.483	

**Figure 2:**
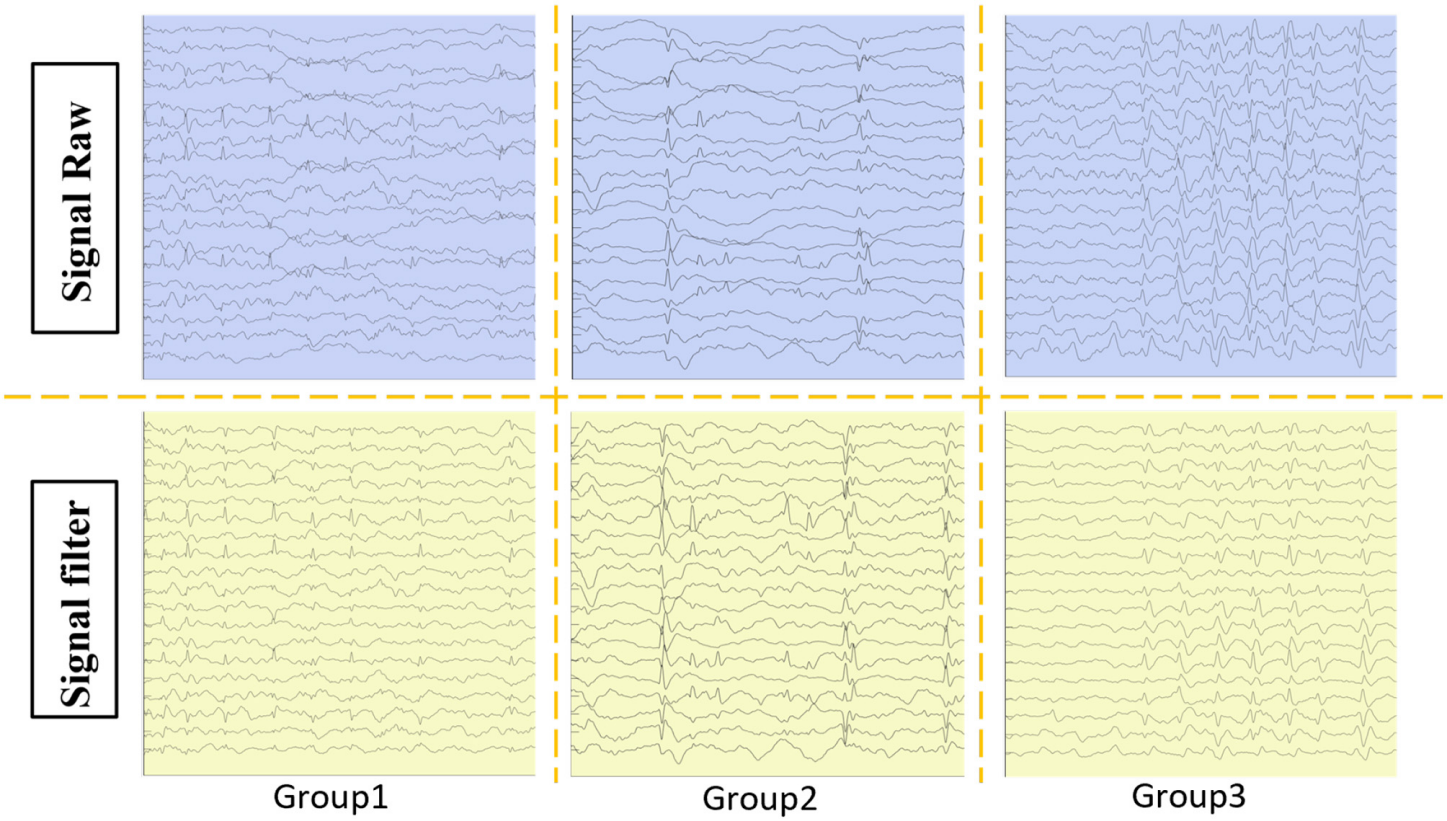
Patients were categorized into three groups based on SWI: group 1: %–65%, group 2: 65%–80%, and group 3: 85%–100%. The raw EEG data from the patients were extracted and filtered.

**Figure 3:**
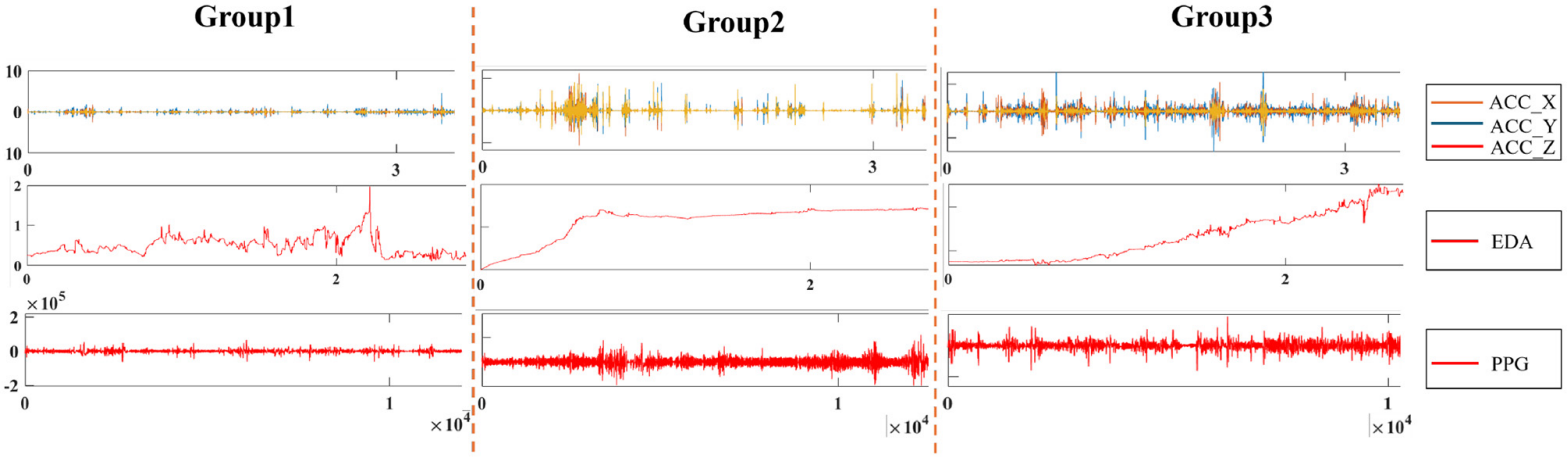
The filtered signals from the ACC, PPG, and EDA in the patient's portable wristband were extracted. PPG, Photoplehysmography; ACC, Three-axis acceleration; EDA, Electrodermal activity.

Figure 2 depicts the discharge patterns during non-rapid eye movement sleep across the three groups, illustrating distinct differences in their EEG activity. Group 3 exhibited greater EEG signal abnormalities than Group 1, where more stable patterns were observed. Figure 3 presents three types of physiological signals: accelerometer signals (*ACC*_*X*, *ACC*_*Y*, *ACC*_*Z*), electrodermal activity (EDA), and photoplethysmography (PPG). The figure demonstrates an overall increasing trend in the amplitude fluctuations of these physiological signals from Group 1 to Group 3. This pattern suggests that Group 3 patients may exhibit more active or unstable physiological states, while Group 1 patients maintain relatively stable physiological conditions. These differences may be related to the grouping criteria (e.g., SWI index) used in this study. Further analysis of the relationship between these physiological signals and clinical indicators could provide deeper insights into patients' disease severity and changes in their physiological state.

### 2.2 Data processing

#### 2.2.1 EEG signal preprocessing and brain network construction

Traditional EEG analysis primarily focuses on changes in electrical activity in individual electrodes or localized brain regions, making it challenging to ascertain a comprehensive picture of the brain's functional state (15) comprehensively. Brain network analysis addresses this challenge by integrating multi-channel EEG signals to construct functional connectivity networks between different brain regions, clearly illustrating information transfer and interaction patterns across them. In SeLECTs patients, abnormal discharges affect local brain regions and may spread to other areas through brain network connections, subsequently exerting widespread impacts on cognitive functions comprehensively (16). Brain network analysis thus facilitates the direct observation of propagation pathways and the extent of abnormal discharges within the brain network, enabling accurate identification of key affected brain regions and their interrelationships.

Channel selection and signal filtering are crucial steps for preprocessing EEG signals. Earlobe electrodes A1 and A2 contribute minimally to the analysis of abnormal discharges in SeLECTs, brain functional network connectivity, and the investigation of cognitive impairment mechanisms. Therefore, their data were removed from the dataset to ensure the brain network analysis focused on core EEG information, improving analysis accuracy and specificity. During data cleaning, a 1–70 Hz filter was applied to prevent the masking of characteristic abnormal discharge signals in SeLECTs by high-frequency electromagnetic interference, ensuring these signals were more distinguishable. Given the prevalence of 50 Hz power-line interference in everyday environments and its significant impact on EEG signal acquisition, a 50 Hz notch filter was employed to eliminate this frequency interference, ensuring that the signals accurately reflected SeLECTs discharge patterns, providing reliable data for constructing brain networks. Additionally, EEG signal accuracy may be compromised by unconscious limb movements during sleep, which can generate motion artifacts. To mitigate this effect, a 500 μV threshold was set for the filtered data, ensuring that the acquired EEG signals more precisely reflect the actual SeLECTs discharge characteristics. This approach enables a more accurate capture of fundamental changes in brain functional connectivity during network construction, providing more reliable evidence for in-depth investigation of the relationship between abnormal discharges and cognitive impairment. δ waves are low-frequency brain waves that are most prominent during the N3 stage of NREM sleep. In patients with ESES, sleep EEG is characterized by continuous spike and slow-wave activity within the 1.5–4 Hz range, which overlaps with the δ wave frequency band. We excluded the alpha frequency band analysis since α rhythms typically dissociate during sleep. Furthermore, considering that hospitalization may negatively affect sleep quality and duration, we focused on the N3 stage of NREM sleep to minimize environmental interference, making delta waves an ideal target for our analysis (6).

Due to variations in EEG sampling duration among SeLECTs patients, the constructed functional brain networks exhibit different sensitivities. Research indicates that longer sampling durations result in reduced functional connectivity values, and different connectivity metrics require varying durations to reach stable states (17). The present study employed the Directed Transfer Function (DTF) method to ensure consistency and accuracy in analyzing abnormal discharge levels and functional connectivity in SeLECTs and further investigate the underlying mechanisms of cognitive impairment. This study employed the Directed Transfer Function (DTF) method. Sleep EEG data from all SeLECTs patients were segmented into 4-s epochs with 50% overlap for subsequent in-depth analysis (18). The DTF method quantifies the direction and strength of information transfer between different nodes in the brain network, enabling a precise analysis of the propagation of abnormal discharges throughout the network and their impact on cognitive function. The DTF calculation formula is generally given by:


$$DTF_{ij}\left(f\right)=\frac{\left|S_{ij}\left(f\right)\right|}{\sqrt{S_{ii}\left(f\right)S_{jj}\left(f\right)}}$$

where *S*_*ij*_(*f*) is an element of the cross-spectral density matrix of multi-channel EEG signals, representing the frequency domain relationship between signals *x*_*i*_(*t*) and *x*_*j*_(*t*).This relationship enables an in-depth exploration of the signal transmission patterns between brain regions associated with abnormal discharges in SeLECTs and their association with cognitive impairment.*S*_*ii*_(*f*) and *S*_*jj*_(*f*) are the power spectral density (PSD) of signals *x*_*i*_(*t*) and *x*_*j*_(*t*), respectively, reflecting the spectral characteristics of each EEG signal. These PSDs help identify the frequency characteristics of abnormal discharges in SeLECTs, facilitating analysis of the impact of these discharges' different frequency components on cognitive function. The magnitude of the cross-spectral density, |*S*_*ij*_(*f*)|, indicates the strength of information transfer from signalis *x*_*i*_(*t*) to *x*_*j*_(*t*). This measure is useful for assessing the propagation strength of abnormal discharges across different brain regions and its relationship with the degree of cognitive impairment. The normalization factor $\sqrt{S_{ii}\left(f\right)S_{jj}\left(f\right)}$ ensures that the DTF values lie within the range [0, 1], allowing for quantitative comparison and analysis of causal relationships between different brain regions.

#### 2.2.2 Preprocessing of multimodal physiological signals from wearable devices

After grouping patients based on SWI criteria, we gave each participant a portable, non-invasive wristband device to investigate its potential as an alternative to long-term video-EEG monitoring. This multimodal physiological signal acquisition system was designed to capture three key biosignals: ACC, EDA, and PPG. Each modality provides multidimensional data support through distinct physiological relevance and sampling frequencies:

- **ACC** signals were collected at a sampling frequency of 50 Hz to capture real-time limb movement states. These signals provided critical data for analyzing movement- interictal epileptiform discharge (IED) relationships, given the established association between limb activity, IEDs during peri-ictal periods, and sleep in SeLECTs patients, as well as their potential cognitive implications.This facilitated exploration of the potential cognitive impacts of abnormal discharges. ACC signal noise primarily originates from physiological movements and motion artifacts (19). To address this, we first removed the 0 Hz component to calibrate baseline drift and eliminate direct current offset effects. Subsequently, a median filter was applied to suppress motion artifacts and high-frequency interference, enhancing signal quality for downstream analysis.
- **EDA** signals were recorded at 4 Hz to monitor autonomic nervous system (ANS) dynamics. During SeLECTs episodes, Sympathetic arousal responses mediated by the ANS have been associated with cognitive alterations during SeLECTs episodes. Continuous EDA monitoring enabled systematic investigation of ANS-cognition interactions (20), providing novel insights into the pathophysiological mechanisms underlying SeLECTs-related cognitive impairment. We implemented a signal quality index (SQI) calculation protocol to ensure data integrity following established methodologies (21).Given EDA signals' slow-varying nature and susceptibility to motion artifacts, we deployed a median filter identical to the ACC processing pipeline to remove contaminating components, improving the specificity of ANS-related electrophysiological features.
- **PPG** signals were acquired at 4 Hz, matching the EDA sampling rate, to provide cardiovascular system metrics. Research has demonstrated significant associations between cardiovascular dynamics, epileptiform discharges, and cognitive dysfunction in SeLECTs. PPG signals therefore offer essential data for investigating cardiovascular-cognition relationships, enabling a systemic perspective on SeLECTs-related cognitive impairment. However, PPG recordings are particularly vulnerable to motion-induced noise contamination. To address this limitation, we applied the same median filtering approach used for ACC and EDA processing, effectively suppressing motion artifacts to ensure reliable signal quality for subsequent cardiovascular feature analysis (22).

Integrating multimodal physiological signals from the wrist-worn device with brain network analysis enables a comprehensive, multidimensional assessment of SeLECTs patients' physiological states and neural functional alterations . This integrated analytical framework provides robust, system-level insights for evaluating cognitive impairment, facilitating mechanistic investigations of the relationship between epileptiform discharges and cognitive dysfunction. To optimize feature extraction and analysis, we rigorously selected 20-min artifact-free recording segments according to strict inclusion criteria (23). These segments were processed using a sliding window approach with a 4-s window duration and a 50% overlap, balancing temporal continuity with independent analytical units (24). Subsequently, modality-specific features were extracted from each window for systematic statistical analysis.

### 2.3 Feature extractions

#### 2.3.1 EEG: extraction of graph theoretic features

Graph theory is a fundamental mathematical framework that represents a practical approach to modeling brain networks. It enables the extraction of critical information about functional brain organization from EEG signals and facilitates the understanding of neural mechanisms in both healthy and pathological states. IIn the present study, we implemented DTF analysis to derive directed graph-theoretical features from SeLECTs patients. This method captures causal relationships and directionality in EEG signals, offering significant insights into propagation patterns and the spatial extent of epileptiform discharges within brain networks (25, 26). Specifically, we focused on analyzing routing and diffusion efficiency to elucidate the potential mechanisms linking abnormal discharges to functional impairments in SeLECTs.

##### 2.3.1.1 Diffusion efficiency

Diffusion efficiency refers to the average first-passage time from node i to node j, reflecting a networks' ability to transmit information between its nodes, where mf pt (i, j) is the mean first passage time from node i to node j.


$$\text{Ediff}\left(i,j\right)=\frac{1}{mf\cdot p_{t}\left(i,j\right)}$$

Global mean diffusion efficiency is the average diffusion efficiency of all node pairs, excluding self-connections. It reflects the overall information transmission and integration capabilities of the brain network.


$$\text{GEdiff}\left(i,j\right)=\frac{\sum_{i\neq j}\text{Ediff}\left(i,j\right)}{n\left(n-1\right)}$$

In this study, diffusion efficiency represents the shortest duration of the transport of information between two channels or brain regions, reflecting the speed of information flow between them.

##### 2.3.1.2 Routing efficiency

Global routing efficiency is an important tool for evaluating the ability of the network to spread information, optimize network structure, and improve network robustness. It results from the sum of the shortest path between all nodes and the average value.


$$\text{GErout}=\frac{\sum \left(\frac{1}{E_{\text{Rout}}\cdot \left(\sim \text{eye}\left(n\right)>0\right)}\right)}{n\left(n-1\right)}$$

In the formula above, *E*_*Rout*_ represents the routing efficiency matrix between nodes. This study defines routing efficiency as the transmission distance of information flow between two channels or brain regions. It reflects the efficiency and reliability of information transmission between these channels or regions. All graph theoretical features in this paper were extracted using the BCT toolbox in MATLAB.

#### 2.3.2 Multimodal physiological signal feature extraction

We implemented a multimodal signal analysis framework to comprehensively investigate physiological state alterations during SeLECTs episodes in multiple dimensions.

- For ACC signals, time-domain features were prioritized due to their computational efficiency, enabling real-time analysis. During SeLECTs episodes, abnormal limb movements were using through ACC time-domain analysis, providing quantitative evidence for examining the impact of epileptiform discharges on motor activity (27).
- PPG signals, reflecting cardiovascular dynamics, were transformed into the frequency domain using Fourier analysis. This enabled the identification of frequency-specific components associated with ictal events (28).
- EDA nonlinear features were analyzed to capture sympathetic nervous system responses. The sensitivity of these features to skin conductance variations enabled precise monitoring of ANS activation during seizures, supporting investigations of ANS cognition interactions (29).

Our feature extraction framework encompassed time-domain, frequency-domain, and nonlinear features, supplemented by statistical descriptors (mean, standard deviation, skewness, and kurtosis) (Table 2) to comprehensively quantify ictal-interictal transitions. This multimodal approach, integrating motor, cardiovascular, and autonomic nervous system metrics, demonstrated superior seizure detection accuracy to unimodal analysis. By correlating these multimodal features with SWI levels, we were able to establish quantitative relationships between electrophysiological markers and physiological responses, advancing our understanding of the mechanisms underlying SeLECTs-related cognitive impairment.

**Table 2:** Signaling characteristic abbreviations.

**Characteristic**	**Abbreviations**
Maximum	Max
Minimum	Min
Peak	Peak
Peak-to-peak	P2P
Mean	Mean
Average amplitude	AVP
Root amplitude	RAT
Standard deviation	SD
Variance	VAR
Root mean squared	RMS
Kurtosis	KUR
Skewness	SKW
Shape factor	SHF
Peaking factor	PKF
Pulse factor	PF
Margin factor	MF
Clearance factor	CF
Mean square frequency	MSF
Frequency component	FC
Root mean square fluctuation	RMSF
Root variance fluctuation	RVF
Mean power spectral density	MPSD
Mean frequency of maximum deviation	MFMD

### 2.4 Statistical analysis

To elucidate the relationships between multimodal signal features, SeLECTs -related seizure activity, and cognitive impairment, we employed rigorous statistical methods, including analysis of variance (ANOVA) and Pearson correlation analysis. The assumptions for the ANOVA were assessed using Shapiro-Wilk normality testing and Levene's test for homogeneity of variance. Multiple comparisons were subsequently performed following significant ANOVA outcomes. The ANOVA was conducted to assess the statistical significance of feature variations across different SWI levels. The F-statistic was calculated as follows:


(1) $$\begin{array}{l} F=\frac{MS_{between}}{MS_{within}} \end{array}$$

where *MS*_*between*_ and *MS*_*within*_ represent between-group and within-group mean squares, respectively. This analysis identified features significantly associated with ictal events, enabling the selection of robust biomarkers for SeLECTs detection.

Pearson correlation analysis quantified the linear relationships between signal features and SWI levels using the following formula:


(2) $$\begin{array}{l} r=\frac{\sum_{i=1}^{n}\left(x_{i}-\bar{x}\right)\left(y_{i}-\bar{y}\right)}{\sqrt{\sum_{i=1}^{n}{\left(x_{i}-\bar{x}\right)}^{2}\sum_{i=1}^{n}{\left(y_{i}-\bar{y}\right)}^{2}}} \end{array}$$

This provided critical insights into feature-severity correlations, supporting the development of accurate association models. The integration of multimodal signals—capturing motor (ACC), cardiovascular (PPG), and autonomic (EDA) dynamics—comprehensively represented physiological states during SeLECTs episodes. This framework significantly enhanced seizure detection accuracy compared to unimodal approaches by leveraging complementary information across physiological systems. By systematically analyzing SWI- dependent variations in multimodal features, we established quantitative association models between electrophysiological markers and physiological responses. These models, parameterized through rigorous statistical analysis, advanced our understanding of the pathophysiological mechanisms underlying SeLECTs-related cognitive dysfunction.

## 3 Results

**Figure 4:**
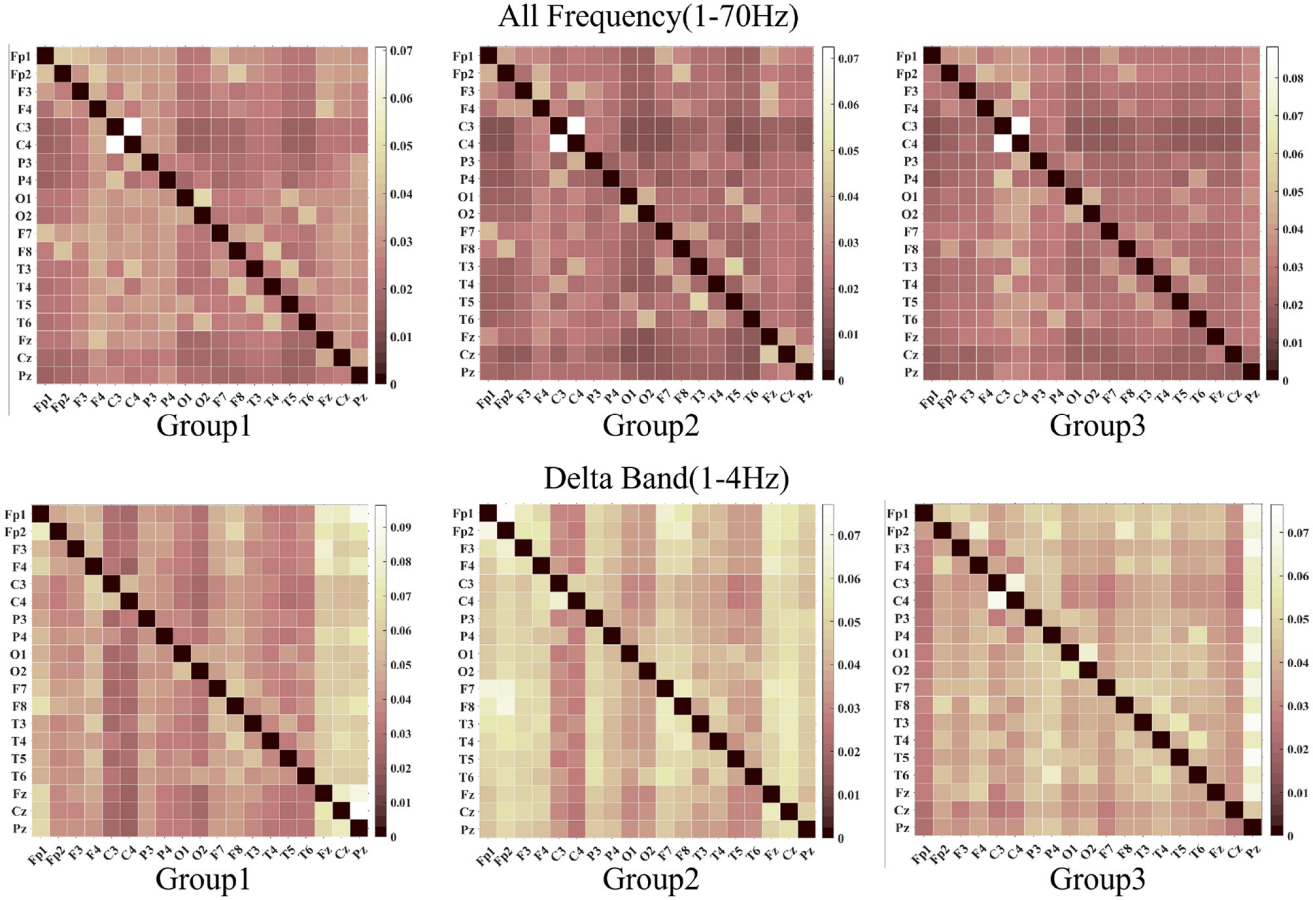
The images above, from left to right, display the visualization results of the DTF function across a frequency range of 1 to 70 Hz. The images below, also from left to right, depict the visualizations for the delta band DTF. In both the delta band and the full-screen segment, the central temporal region exhibits strong functional connectivity.

**Figure 5:**
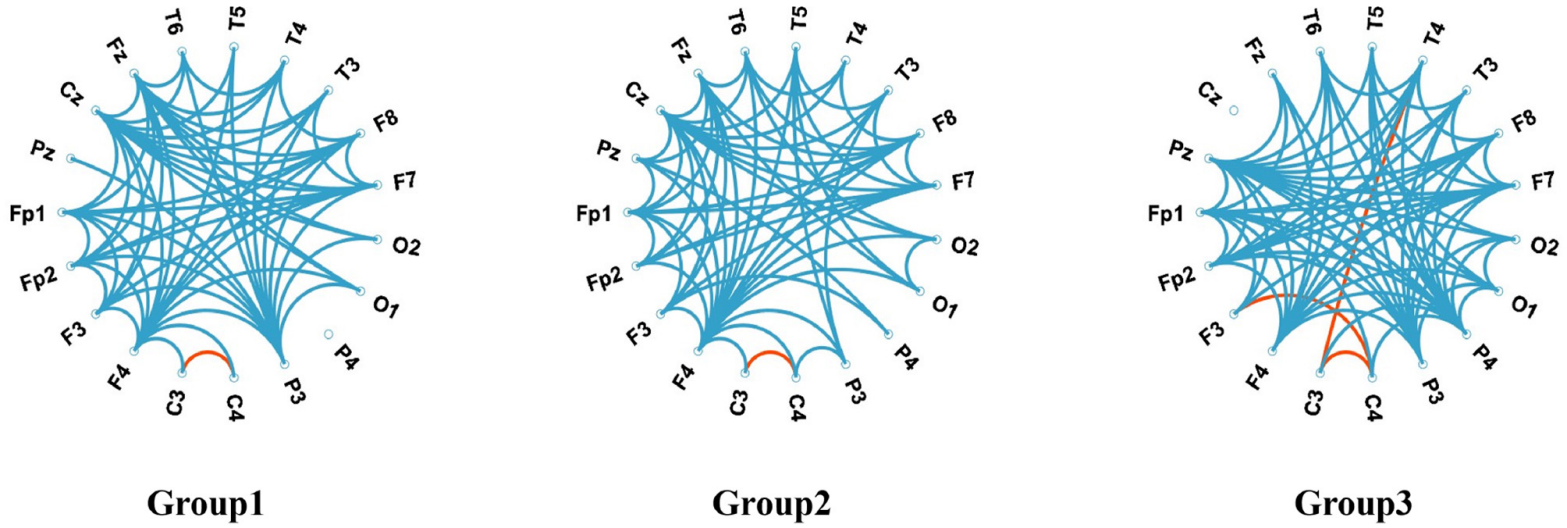
Chordal maps illustrate the differences in brain network connectivity. Blue lines indicate that Delta bands exhibit reduced functional connectivity compared to the full-screen segment bands. In contrast, red lines show that Delta bands have increased functional connectivity compared to the full-screen segment bands.

We employed DTF to characterize brain network dynamics for functional connectivity visualization and analysis, as shown in Figures 4, 5. The whole frequency band (1–70 Hz) and delta band (1–4 Hz) exhibited enhanced functional connectivity in the centrotemporal regions, reflecting increased neuronal synchronization associated with epileptiform discharges. Comparative analysis revealed significant between-group differences in connectivity patterns, demonstrating the influence of SWI levels on interregional functional organization. Graph-theoretical analysis further indicated that higher SWI levels were associated with decreased network diffusion and routing efficiency.

**Figure 6:**
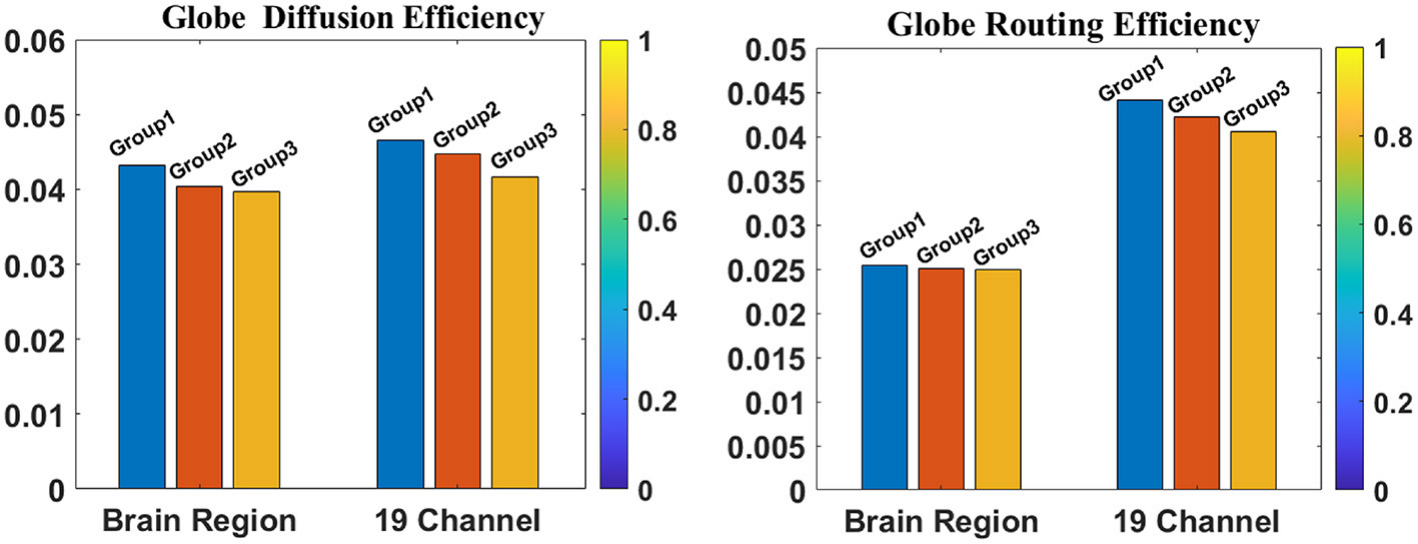
This figure illustrates two theoretical concepts in graph theory: diffusion efficiency and routing efficiency. The **left panel** displays diffusion efficiency, while the **right panel** shows routing efficiency.

We conducted a detailed analysis of graph-theoretical metrics, focusing on diffusion and routing efficiency of SeLECTs activity, as illustrated in Figure 6. Participants were stratified into three groups based on SWI levels: Group 1 (50%–65%), Group 2 (65%–80%), and Group 3 (85%–100%). The analysis revealed an inverse relationship between SWI levels and network efficiency metrics, with both diffusion and routing efficiency demonstrating significant declines as SWI increased. This pattern indicates that heightened epileptiform discharges impair the brain's capacity for information propagation and integration (30). The observed network inefficiencies may underlie specific cognitive deficits, particularly in attention, memory, and executive functioning.

The results of the multidimensional feature analysis of the ACC, PPG, and EDA signals are shown in Table 3. The analysis indicates that features such as Maximum, Minimum, and Peak in the ACC and PPG all exhibit significant differences under different experimental conditions(*P* < 0.05). The changes in these features may reflect the abnormal limb movement patterns of patients during SeLECTs seizures and the disorder of the autonomic nerve regulation function of the cardiovascular system (31). It is worth noting that the analysis results of the EDA signal show that except for Variance and Root Mean Square Fluctuation, the remaining features all show significant statistical differences (*P* < 0.05), and the magnitude of their changes is significantly larger than that of the ACC and PPG signals.

**Table 3:** *p*-values of different features on 3-modal signals.

**Features**	**ACC**	**PPG**	**EDA**
Maximum	0.0469	0.0120	0.0430
Minimum	0.0465	0.0110	0.0248
Peak	0.0065	0.0200	0.0443
Peak-to-Peak	0.0091	0.0031	0.0486
Mean	0.0353	0.0362	0.0424
Average amplitude	0.0258	0.0063	0.0441
Root amplitude	0.0264	0.0483	0.0371
Standard deviation	0.0211	0.0356	0.0088
Variance	0.0122	0.2933	0.0096
Root mean squared	0.0057	0.0014	0.0085
Kurtosis	0.0011	0.0012	0.0041
Skewness	0.0261	0.0168	0.0208
Shape factor	0.0035	0.0030	0.0021
Peaking factor	0.0201	0.0013	0.0026
Pulse factor	0.0012	0.0011	0.0021
Margin factor	0.0209	0.0448	0.0201
Clearance factor	0.0119	0.0168	0.0094
Mean square frequency	0.0060	0.0065	0.0031
Frequency component	0.0015	0.0050	0.0072
Root mean square fluctuation	0.0015	0.0041	0.8600
Root variance fluctuation	0.0312	0.0355	0.0186
Mean power spectral density	0.0028	0.007	0.0018

The assumptions for ANOVA were assessed using Shapiro-Wilk normality testing and Levene's test for homogeneity of variance.

**Figure 7:**
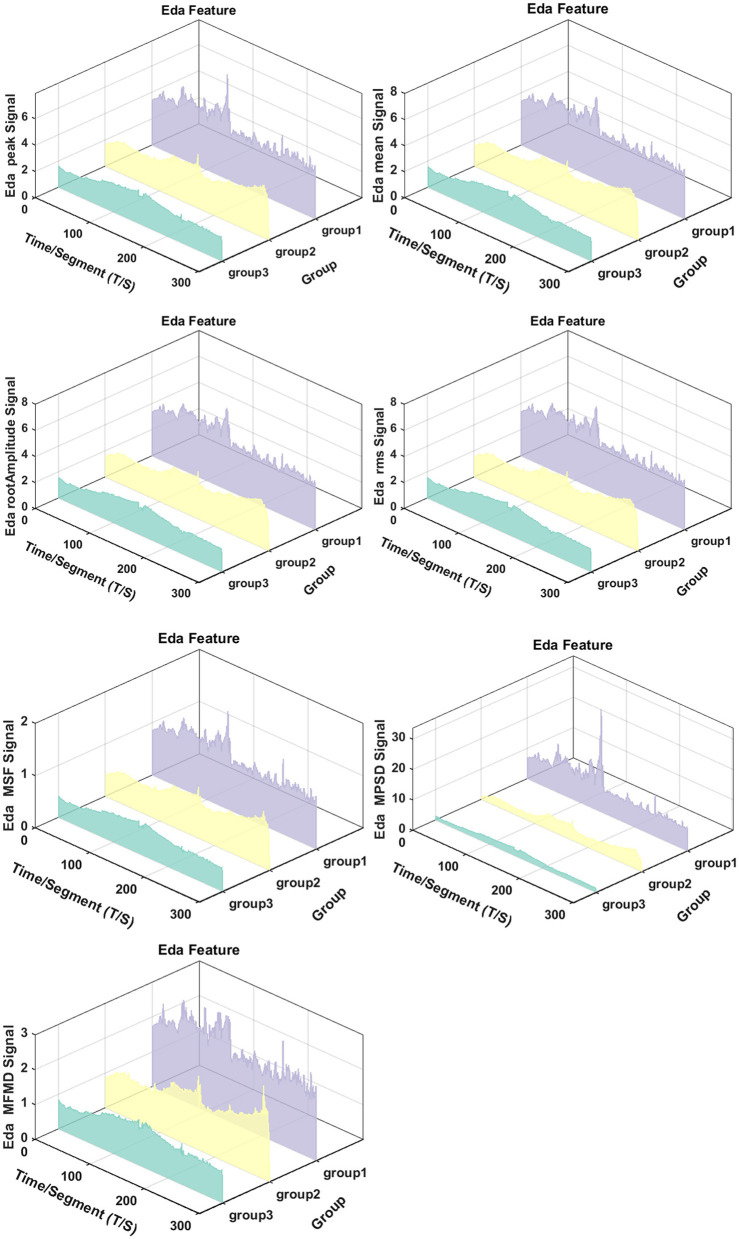
The figure reveals significant intergroup differences in the spatiotemporal dynamics of EDA. From left to right, the features include EDA Peak, EDA Mean, EDA Root Amplitude, and EDA RMS in the time-domain, as well as EDA MSF, EDA MPSD, and EDA MFMD in the frequency-domain. Peak, Peak; Mean, Mean; RAT, Root Amplitude; RMS, Root Mean Squared; MSF, Mean Square Frequency; MPSD, Mean Power Spectral Density; MFMD, Mean Frequency of Maximum Deviation.

We analyzed both time-domain (EDA Peak, EDA mean, EDA root Amplitude, EDA RMS) and frequency-domain (EDA MSF, EDA MPSD, EDA MFMD) features of EDA across different SWI groups, as shown in Figure 7. Significant between-group differences were observed in EDA temporal dynamics. For instance, EDA Peak values were consistently higher in Group 1 compared to Group 3 during specific time intervals, with statistically significant differences across multiple epochs. These findings suggest a systematic relationship between EDA features and SWI levels, indicating suppressed sympathetic nervous system activity in patients with higher SWI.

## 4 Discussion

### 4.1 Analysis of neurophysiological signals in the brain function impairment mechanism caused by SeLECTs and high spike-wave index

This study investigated the neurophysiological mechanisms underlying brain functional impairment in SeLECTs using EEG analysis. Our findings demonstrate a significant inverse relationship between SWI and brain network efficiency metrics, notably global and local efficiency. The observed reductions in diffusion efficiency (reflecting information propagation speed) and routing efficiency (indicating optimal path selection) suggest that SeLECTs patients exhibit impaired information transfer and integration. These network inefficiencies may lead to slowed or unstable interregional communication, providing a neurophysiological basis for the cognitive deficits—particularly in executive functioning and memory processing—observed during SeLECTs patients' active seizure periods. Our analysis revealed dynamic, SWI-dependent alterations in functional connectivity and graph-theoretical metrics, indicating a dose-response relationship between epileptiform discharge intensity and network dysfunction. At lower SWI levels, the brain network retains partial information transfer efficiency; however, as SWI increases, the disruptive effects of abnormal discharges progressively intensify, ultimately leading to significant network efficiency degradation. These findings suggest that SeLECTs patients exhibit a state of network instability, in which discharge intensity and frequency directly modulate network stability and efficiency. Consequently, clinical management strategies should extend beyond seizure control to include targeted network restoration and stabilization interventions, potentially improving cognitive outcomes in affected patients.

### 4.2 Applications of non-invasive wristband devices

A key objective of this study was to develop an accurate and convenient alternative to long-term video-EEG monitoring using portable devices. Leveraging the technical capabilities of OPPO watch sensors, we investigated PPG, EDA, and ACC signals as primary monitoring metrics. Our results demonstrated the superior performance of EDA in detecting SeLECTs -related ANS alterations, exhibiting greater sensitivity and dynamic range than ACC and PPG signals. Specifically, several key EDA parameters, such as peak value, root amplitude, RMS, temporal mean value, mean square frequency, average power spectral density, and maximum deviation of mean frequency, all demonstrated significant reductions. These findings align with Horinouchi et al.'s (32) reports of EDA attenuation in epilepsy patients due to autonomic dysfunction, providing additional objective evidence of peripheral autonomic abnormalities in epilepsy.

From a neurophysiological perspective, elevated SWI may reflect the pathological hypersynchronization of cortical discharges during epileptic episodes (33). This excessive synchronization could impair information integration and transmission efficiency among neural network nodes, potentially affecting higher cognitive functions such as attention and memory. Concurrently, the reduction in EDA signals might result from descending inhibition of the peripheral autonomic nervous system by epileptic activity. Research indicates that epileptiform discharges are not confined to the central nervous system but can also suppress preganglionic sympathetic neuronal activity through descending pathways, including the brainstem reticular formation, leading to diminished skin conductance responses. More significantly, increased SWI may disrupt the negative feedback regulation of the hypothalamic-pituitary-adrenal (HPA) axis, altering the rhythmic secretion of glucocorticoids (34). This disruption could further suppress sympathetic nervous system excitability, reducing EDA signal intensity. The bidirectional interaction between the central and peripheral ANS likely forms the neurobiological basis for characteristic EDA signal alterations in SeLECTs patients.

It should be noted that while elevated SWI levels may correlate with increased seizure frequency, currently, there is no direct evidence establishing a causal relationship between increased SWI and decreased EDA signal characteristics. However, it should be noted that while elevated SWI levels may correlate with increased seizure frequency, current research has not provided direct evidence establishing a causal relationship between increased SWI and decreased EDA signal characteristics. Vieluf et al. (35) observed circadian rhythm alterations in EDA patterns among epilepsy patients during 24-h continuous monitoring. These alterations were characterized by overall reductions in skin conductance level and amplitude of the skin conductance response. A preceding study demonstrated that EDA has the capacity to reduce the frequency of seizures and is associated with widespread activation of the cerebral cortex (36, 37). This study further corroborated the findings that EDA signals are significantly linked to cerebral network activity and revealed alterations in specific network characteristics. These findings are of particular significance for patients who experience seizure warning symptoms or prodromal symptoms prior to seizures, as they provide a basis for implementing timely preventive strategies. Furthermore, the study indicated that SWI levels may play a substantial role in these autonomous changes. This finding reveals a complex interaction between central and peripheral autonomous regulation in epilepsy. This objective evidence is pivotal for clinical diagnosis and understanding of epilepsy-related autonomous nervous system dysfunction. EDA signal monitoring has the capacity to facilitate real-time seizure activity assessment, facilitate identification of high-risk seizure states (ESES), and enhance the refinement of treatment and follow-up management plans.

### 4.3 Study limitations and future directions

This study contributes to the understanding of brain function impairment mechanisms in SeLECTs by applying neurophysiological signal analysis. It explores a potential alternative to long-term video—EEG monitoring using portable devices. However, several limitations should be acknowledged, and future research directions are proposed to advance this field. Our study recognizes several important limitations in our participant selection process: (1) the lack of a control group that includes both non-ESES epileptic patients (e.g., patients with focal epilepsy with SWI <50%) and healthy controls. Although the 85% threshold has historically been dominant, recent guidelines suggest a lower threshold of SWI ≥50% in NREM sleep. However, some groups have adopted thresholds as low as 25%, and thus these categorization criteria contributed to our SWI cut-off values and the analysis of the control group (5, 38). (2) Relatively small sample size. Although our research strategy is consistent with previous ESES literature (39–41), these observations should be interpreted with caution, and may not be generalizable.These limitations primarily reflect the practical challenges of conducting a single-center exploratory study with stringent inclusion criteria: all enrolled ESES patients were required to meet both rigorous electroclinical diagnostic standards (SWI ≥85%) with documented cognitive regression) and complete multimodal neurophysiological assessments, which significantly prolonged case recruitment and data collection timelines. We plan to collaborate on future multicenter studies where the inclusion of diverse comparison cohorts will be more feasible.

The current classification framework implicitly acknowledges the phenotypic continuum of epilepsy, where some cases may transition from benign presentations to non-benign stages during disease progression, potentially accompanied by cognitive decline (2, 3). However, while these systems theoretically recognize this continuum, further clinical validation is required to clarify critical parameters. These include the specific proportion of children with SeLECTs who progress to ESES, develop language or cognitive regression, or evolve into drug-resistant epilepsy, as well as the predictive factors underlying such transitions. More than 50 years since the first description of ESES, the pathophysiologic mechanisms underlying the emergence of encephalopathies associated with enhanced sleep-related epileptic discharges remain incompletely elucidated (42). Current research on SWI and cognitive impairment remains scarce. The effect of spikes on long- and short-term cognitive impairment remains unclear. The present study's findings could be further strengthened by optimizing data acquisition and analysis methods for non-invasive wristband devices, particularly in terms of enhancing their accuracy and convenience for epilepsy monitoring. Future studies should consider expanding the sample size and implementing multicenter research designs to validate the generalizability and reliability of the results. Additionally, extending monitoring periods would enable researchers to capture dynamic changes in physiological signals, potentially leading to the development of comprehensive datasets for early disease warning systems and facilitating personalized treatment strategies using through longitudinal data analysis (43). These future directions would address current limitations while building upon the foundation established by this study, potentially leading to more effective monitoring and treatment approaches for SeLECTs patients.

## 5 Conclusions

This study aimed to investigate the intrinsic neural mechanisms underlying brain function impairment caused by abnormal discharges SeLECTs and to explore the potential of non-invasive wristband devices in epilepsy monitoring. The results demonstrate that increased SWI in SeLECTs patients significantly correlates with reduced global and local efficiency of brain functional networks, revealing the detrimental effects of abnormal discharges on brain information transmission and integration capabilities. Simultaneously, monitoring through non-invasive wristband devices revealed that EDA signals exhibit excellent performance in capturing SeLECTs-related autonomic nervous system activity changes. The observed decrease in EDA parameters shows a strong association with both central nervous system abnormal discharges and peripheral autonomic inhibition, further elucidating the bidirectional interactions between central and peripheral nervous systems. These findings provide novel insights into the pathological mechanisms of SeLECTs and establish a foundation for optimizing and clinically applying non-invasive monitoring technologies. Future research should focus on expanding sample sizes, prolonging monitoring durations, and refining analytical methods to advance early warning systems and personalized treatment strategies for SeLECTs.

## Data Availability

The raw data supporting the conclusions of this article will be made available by the authors, without undue reservation.
