# Correction: Study on brain functional networks and wearable device monitoring in children with SeLECTs and high spike-wave index (SWI >50%)

**DOI:** 10.3389/fneur.2025.1719076

**Published:** 2025-10-21

**Authors:** Minghao Xu, Xiaoxuan Li, Yifan Fu, Dinghan Hu, Yang Zhang, Wang Wei, Yaotian Gao, Keyi Lin, Bin Yang, Jiuwen Cao, Tao Jiang

**Affiliations:** ^1^Department of Neurosurgery, The First Affiliated Hospital of Anhui Medical University, Hefei, Anhui, China; ^2^Anhui Public Health Clinical Center, Hefei, Anhui, China; ^3^Machine Learning and I-Health International Cooperation Base of Zhejiang Province, and the Artificial Intelligence Institute, Hangzhou Danzi University, Zhejiang, China; ^4^Department of Neurosurgery, The First Affiliated Hospital of Anhui University of Traditional Chinese Medicine, Hefei, Anhui, China; ^5^Department of Neurology, Anhui Province Children's Hospital, Hefei, Anhui, China

**Keywords:** SWI, ACC, PPG, EDA, DTF, SeLECTs

In the published article, affiliation 2, ‘Anhui Public Health Clinical Center, Hefei, Anhui, China’ was omitted for authors Minghao Xu and Tao Jiang. This affiliation has now been added for both authors to include both affiliation 1 ‘Department of Neurosurgery, The First Affiliated Hospital of Anhui Medical University, Hefei, Anhui, China, and affiliation 2 ‘Anhui Public Health Clinical Center, Hefei, Anhui, China’ for authors Minghao Xu and Tao Jiang.

The original version of this article has been updated.

